# The bromodomain inhibitor IBET-151 attenuates vismodegib-resistant esophageal adenocarcinoma growth through reduction of GLI signaling

**DOI:** 10.18632/oncotarget.27699

**Published:** 2020-08-18

**Authors:** Annamil Alvarez-Trotta, Zhiqiang Wang, Elena Shersher, Bin Li, Jun Long, Ines Lohse, Claes Wahlestedt, Wael El-Rifai, David J. Robbins, Anthony J. Capobianco

**Affiliations:** ^1^Molecular Oncology Program, Division of Surgical Oncology, Dewitt Daughtry Family Department of Surgery, University of Miami, Miami, FL, USA; ^2^Sylvester Comprehensive Cancer Center, Miller School of Medicine, University of Miami, Miami, FL, USA; ^3^Division of Surgical Oncology, DeWitt Daughtry Family Department of Surgery, Miller School of Medicine, University of Miami, Miami, FL, USA; ^4^Center for Therapeutic Innovation, Miller School of Medicine, University of Miami, Miami, FL, USA; ^5^Department of Psychiatry and Behavioral Sciences, Miller School of Medicine, University of Miami, Miami, FL, USA; ^6^Molecular Therapeutics Shared Resource, Sylvester Comprehensive Cancer Center, University of Miami, Miami, FL, USA; ^*^Co-senior authors

**Keywords:** IBET-151, esophageal adenocarcinoma, GDC-0449 resistance, patient-derived xenografts, Hedgehog/GLI signaling pathway

## Abstract

The Hedgehog/GLI (HH/GLI) signaling pathway plays a critical role in human oncogenesis. Unfortunately, the clinical use of HH inhibitor(s) has been associated with serious adverse effects and mutation-related drug resistance. Since the efficacy of SMO (Smoothened) and GLI inhibitors is limited in clinical trials, there remains a critical need for the HH/GLI pathway inhibitors with different mechanisms of action. Here, we show that esophageal adenocarcinoma (EAC) cell lines are insensitive to vismodegib (SMO inhibitor) but respond to GANT61 (GLI1 inhibitor). Furthermore, we examine the role of GLI1 in tumorigenicity of EAC and how a selective bromodomain inhibitor IBET-151 downregulates transcriptional activity of the GLI1 transcription factor in EAC. Our study demonstrates that GLI1 plays an important role in tumorigenicity of EAC and that elevated GLI1 expression in patients’ ultrasound-assisted endoscopic biopsy may predict the response to neoadjuvant chemotherapy (NAC) FOLFOX. Importantly, IBET-151 abrogates the growth of vismodegib-resistant EAC cells and downregulates HH/GLI by reducing the occupancy of BRD4 at the GLI1 locus. IBET-151 also attenuates tumor growth of EAC-PDXs and does so in an on-target manner as it reduces the expression of GLI1. We identify HH/GLI signaling as a novel druggable pathway in EAC as well as validate an ability of clinically relevant GLI inhibitor to attenuate the viability of vismodegib-resistant EAC cells. Therefore, we propose that selective bromodomain inhibitors, such as IBET-151, could be used as novel therapeutic agents for EAC patients harboring GLI-dependent tumors.

## INTRODUCTION

Esophageal adenocarcinoma (EAC) is one of the most aggressive cancers in the world that is characterized by a high mortality rate and poor prognosis [1]. The incidence of EAC has been on the rise in the United States and other western countries over the past 30 years [2]. Despite multidisciplinary therapeutic approaches, EAC remains a virulent disease with an overall 5-year survival rate < 20% [1, 2]. There is a great urgency to develop more effective treatment strategies in order to improve clinical outcomes. Recent studies have suggested that constitutive activation of the HH pathway in cancers of the digestive tract may contribute to the growth and maintenance of cancer [3]. However, the relationship between HH signaling and therapeutic response is unknown. The HH pathway and associated overexpression of GLI1 have been reported as oncogenic [4] while the nuclear expression of GLI1 is considered predictive of a pathologic complete response to chemoradiation in esophageal cancer (EC) [5]. Although there have been some advances in the discovery of molecular drivers of EC, a detailed understanding of the molecular mechanisms that promote EAC progression is still limited.

HH signaling pathway plays a critical role in regulating both embryonic development and cancer [6]. The HH signaling network comprises both canonical and non-canonical signaling pathways. Activation of canonical HH signaling occurs when any of the three HH ligands binds to its receptor complex, which includes the pivotal negative regulator Patched (PTCH) [6]. This relieves the repression of SMO by PTCH, which ultimately leads to the activation, stabilization, and nuclear translocation of the GLI family of transcription factors. In humans, there are three different GLI proteins (GLI1, GLI2, and GLI3). While GLI1 is exclusively a transcriptional activator, GLI2 and GLI3 can function as both transcriptional activators and repressors [6–8]. To date, numerous GLI target genes have been described, which are involved in feedback mechanisms (e.g., *HHIP, PTCH1, GLI1*), cell cycle regulation (e.g., *CYCLIN D1/2*), proliferation, (e.g., *PDGFR, MYC*) apoptosis (e.g., *BCL2*), angiogenesis (e.g., *VEGF, ANG1/2*), epithelial-mesenchymal transition (EMT; e.g., *MMP9, SNAIL*) and self-renewal (e.g., *NANOG, SOX2*), representing a broad spectrum of mechanisms by which the HH signaling pathway can be involved in carcinogenesis [6–9]. SMO is the main transducer of the HH signaling pathway. Accordingly, SMO inhibitors have received intense research attention since the identification of cyclopamine (a natural steroidal alkaloid) as the first SMO antagonist, which blocks the HH signaling pathway [10]. Notably, vismodegib (GDC-0449) [11] and sonidegib (LDE225) [12] were approved by the US Food and Drug Administration (FDA) for the treatment of basal cell carcinoma (BCC) in 2012 and 2015, respectively. Several other SMO inhibitors have also moved into various stages of clinical trials [13]. Unfortunately, the clinical use of SMO inhibitor (s) has also been associated with adverse effects and mutation-related drug resistance. Subsequent studies of acquired resistance to SMO inhibitors suggested possible mechanisms to explain this resistance: (i) a mutation in SMO that prevents ligand binding; (ii) the upregulation of downstream effectors in the HH signaling cascade (such as GLI); and (iii) activation of oncogenic signaling pathways that interact with HH [13, 14]. For example, it has been reported that GLI1 can participate in a crosstalk with the mTOR pathway to induce secondary resistance to HH inhibition in EC [15]. Since the GLI proteins are the final effectors of HH pathway, the development of GLI-targeted approach would be useful for downregulating both canonical and non-canonical HH pathway activation and perhaps overcoming anti-SMO drug resistance.

Inhibition of BET (bromodomain and extra-terminal motif) bromodomain proteins has recently emerged as a novel strategy to epigenetically target the HH pathway transcriptional output [16]. The BET bromodomain protein BRD4 is a critical regulator of *GLI1* and *GLI2* transcription via direct occupancy on their promoters [16, 17]. Previously, we reported a BET inhibitor (IBET-151) as a specific modulator of HH signaling that acts downstream of SMO [18]. Our new results show that aberrant activation of GLI signaling is observed in EAC cell lines and primary patient-derived tumors, and the expression level of GLI1 is associated with the clinical stage of EAC. Moreover, we observed that GLI1 may predict the response to neoadjuvant chemotherapy (NAC-FOLFOX). Therefore, we hypothesize that HH/GLI pathway is critical in tumorigenicity of EAC. Furthermore, we show that IBET-151 abrogates the growth of vismodegib-resistant EAC cells by reducing the occupancy of BRD4 at the *GLI1* locus that results in HH/GLI downregulation. IBET-151 also attenuates tumor growth of EAC-PDXs. Together, our results identify a novel druggable signaling pathway in EAC and confirm the ability of a clinically relevant GLI inhibitor to attenuate EAC growth.

## RESULTS

### Elevated GLI1 activity, associated with the differentiation and clinical stage of EAC, drives resistance to chemotherapy

**Figure 1:**
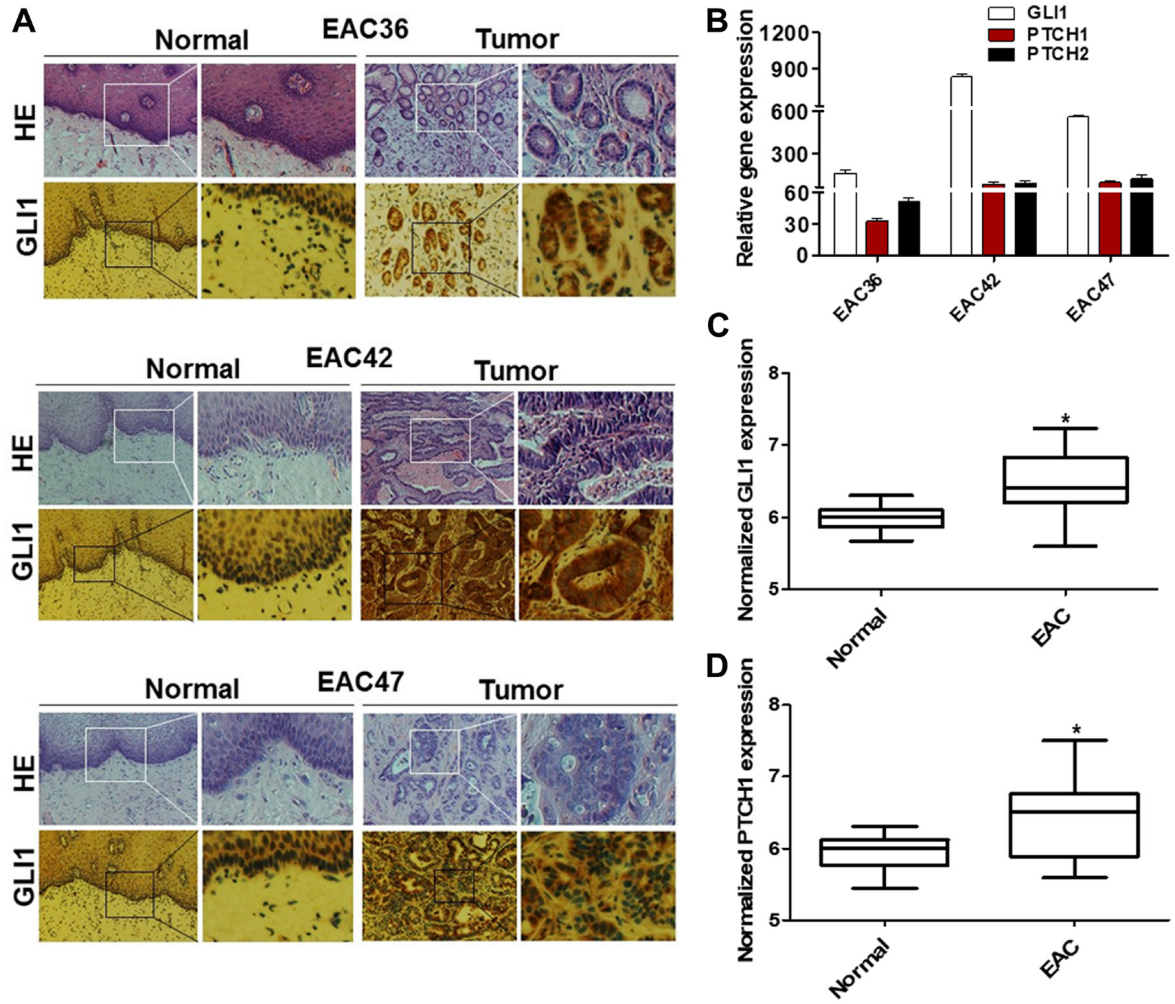
GLI1 activity is increased in human EAC tumors. (**A**) Hematoxylin and eosin (H&E) and IHC staining of GLI1 were determined in primary human EAC tumors (EAC36, EAC42, and EAC47) and matched adjacent normal mucosa. EAC tumors show strong nuclear staining for GLI1 while only cells in the basal layer of the normal esophageal mucosa exhibited faint staining for GLI1. (**B**) mRNA levels of HH target genes were observed in primary human esophageal tumor and were normalized to the relative expression values of matched adjacent normal mucosa (*N* = 3). Error bars represent the S. E. of three independent experiments. Boxplot depicting mRNA levels for GLI1 (**C**) and PTCH1 (**D**) genes from patients’ samples EAC tumors (*n* = 75) and adjacent normal mucosa (*n* = 28) were analyzed from TCGA RNAseq. Y-axis show counts normalized by DESeq statistical method. *p* values ≤ 0.05 are considered statistically significant and indicated by an asterisk (^*^).

**Figure 2:**
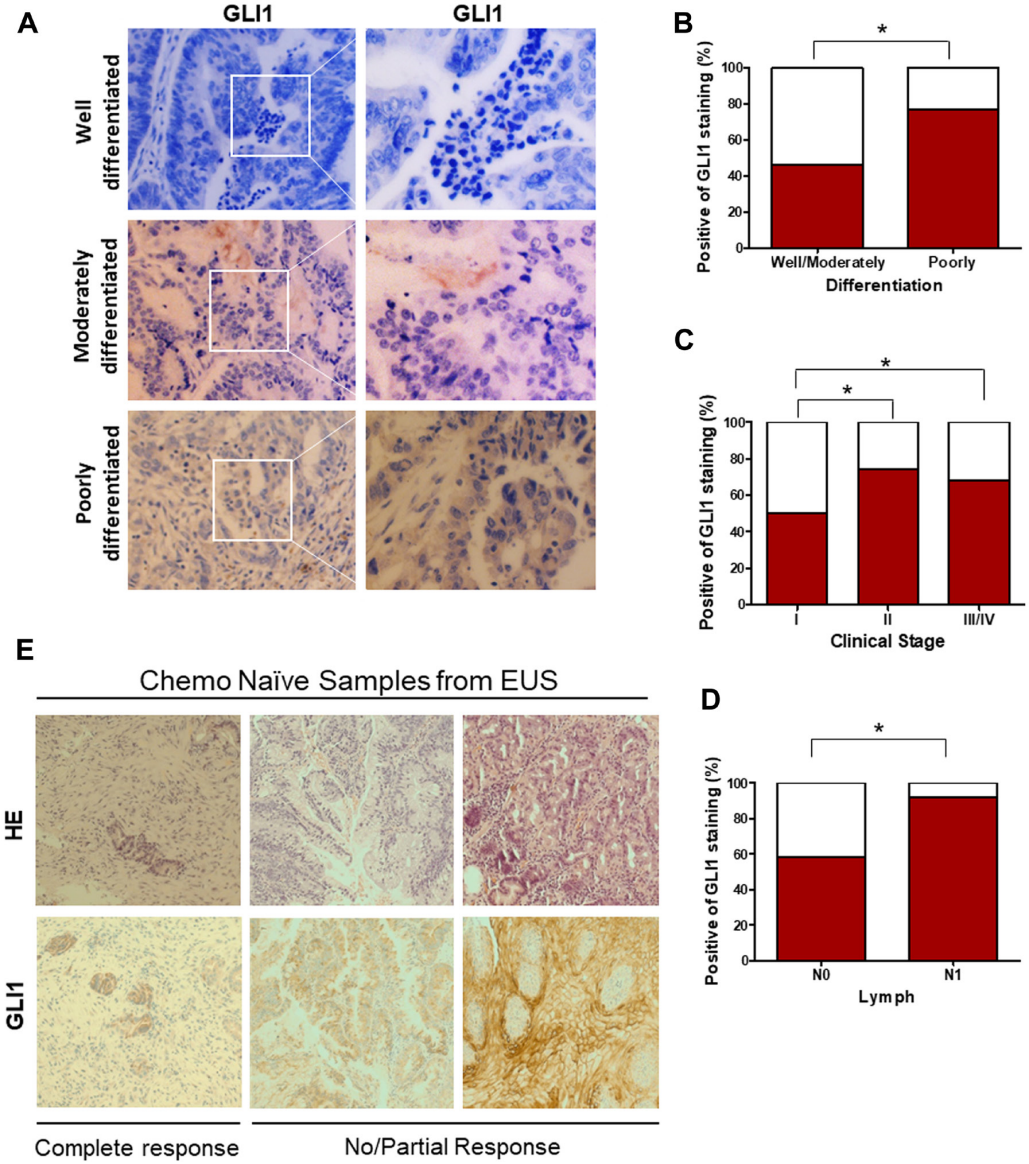
Elevated GLI1 activity is associated with the differentiation state and clinical stage of EAC, drives resistance to the chemotherapy. (**A**) IHC staining of GLI1 was determined in the EAC with well-, moderately-, and poorly-differentiated tumors. (**B**) Percent positive of GLI1 staining in the EAC with well-, moderately-, and poorly-differentiated tumors. (**C**) Percent positive of GLI1 staining in the EAC with different clinical stages. (**D**) Percent positive of GLI1 in EAC with lymph node metastasis (N0: 0 lymph node, N1:1–3 axillary lymph nodes +). (**E**) H&E and IHC staining of GLI1 in human esophageal ultrasound-assisted (EUS) biopsies. (C) *p* values ≤ 0.05 are considered statistically significant and indicated by an asterisk (^*^).

In order to directly assess the status of GLI signaling in patient-derived EAC samples, we screened primary normal esophageal mucosa and EAC cells for presence of GLI1 using immunohistochemistry (IHC) (Figure 1A). Significant positive levels of GLI1 were present in 62% (37/60) of primary EAC tumor tissues. In contrast, mild levels of GLI1, localized to cells within the basal layer, were detected in 17% of normal esophageal mucosa samples. Next, we compared the expression levels of various HH target genes (*GLI1, PTCH1*, and *PTCH2)* in normal human primary esophageal epithelial cells and EAC cell lines derived from tumor samples (Figure 1B). All three target genes were expressed at significantly higher levels in the primary EAC tumors relative to normal mucosa. Gene expression data of 103 patient samples (75 EAC tumors and 28 adjacent normal mucosa) from the Cancer Genome Atlas (TCGA) were also analyzed. We selected target genes commonly used to identify the GLI-dependent HH signaling activation, such as *GLI1, GLI2, PTCH1, HHIP,* and *MYCN*. The expression of this larger panel of HH target genes was significantly higher in EACs than in normal tissue (Figure 1C and 1D; Supplementary Figure 1A–1C). Furthermore, GLI1 levels appeared to vary depending on the degree of tumor cell differentiation (Figure 2A). Strongly positive levels of GLI1 were observed in 77% (22/28) of poorly-differentiated EAC tumors, whereas mild positive nuclear staining for GLI1 was observed in 46% (15/32) of the well and moderately differentiated EAC cases (Figure 2B) We also determined that GLI1 levels vary depending on the clinical stage (Figure 2C) and lymph node metastasis of EAC tumors (Figure 2D), increasing with the stage of the disease. This indicates that GLI1 activity correlates with the stage of EAC diagnoses. Together, these results suggest that GLI signaling is aberrantly activated in EAC. Current treatment guidelines for EAC include NAC followed by surgical resection [19–21]. In order to determine whether GLI1 played a role in the response to NAC FOLFOX, we analyzed the expression of GLI1 in a set of chemo-naive EAC samples derived from ultrasound-assisted endoscopic biopsies and determined its correlation to the pathological response to NAC. Twelve out of 22 patient tumors that had undetectable levels of GLI1 had a complete response to chemotherapy, whereas ten patients that had high or moderate levels of GLI1 expression did not show significant response to chemotherapy (Figure 2E). Thus, elevated expression of GLI1 appears to predict tumor response to chemotherapy and is associated with poor prognosis in EAC.

### Gli1 is a critical regulator of EAC viability

**Figure 3:**
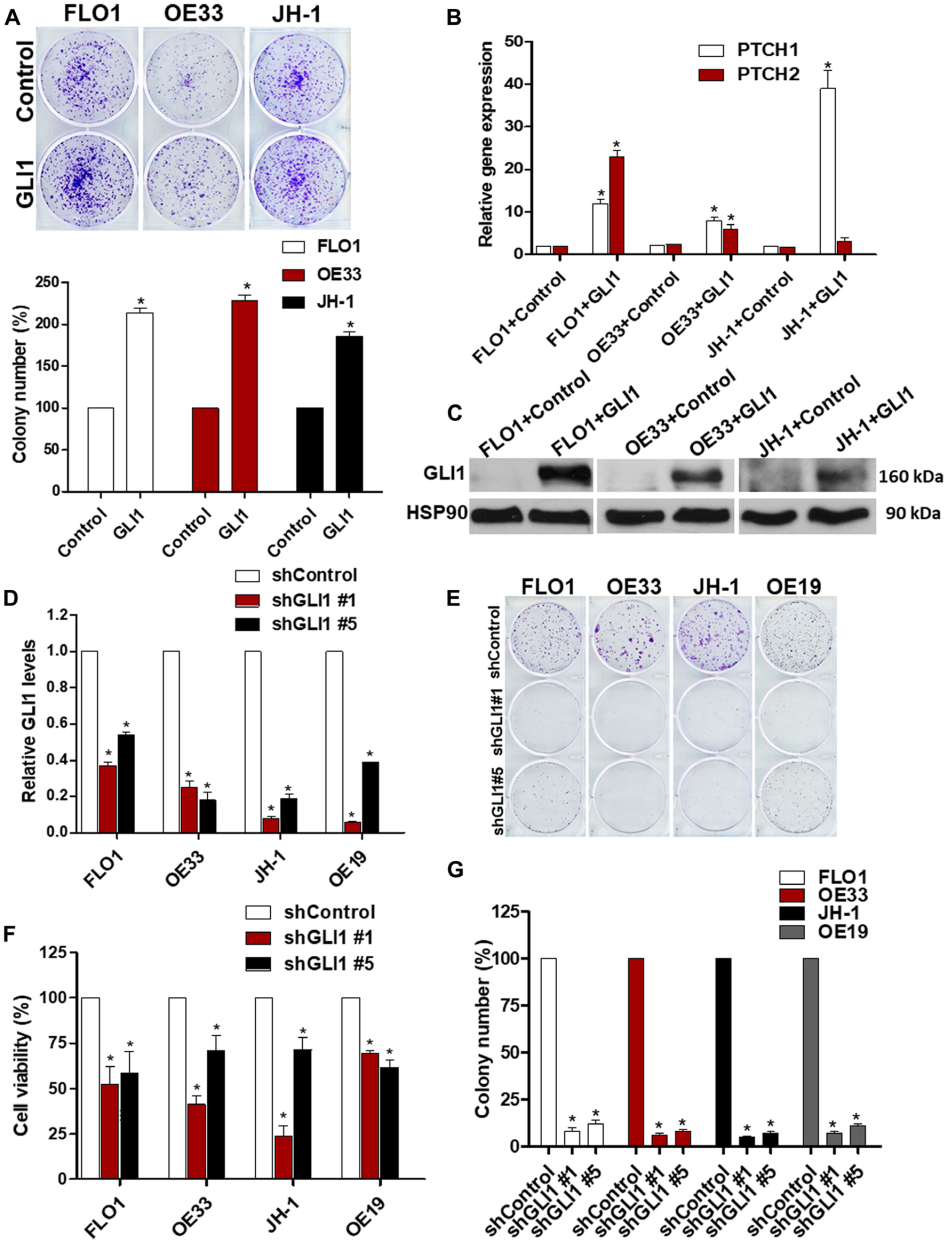
GLI1 is a critical regulator of EAC viability. (**A**) Ectopic GLI1 increases the ability of EAC cells to grow in a colony assay. The quantification of the colony number of EAC cell lines under ectopic expression of GLI1 was normalized to the control (*N* = 3). (**B**) mRNA levels of GLI1 target genes (*PTCH1* and *PTCH2*) were observed in EAC cell lines under ectopic expression of GLI1 and were normalized to the relative expression values of the control (*N* = 3). (**C**) The levels of GLI1 protein expression were analyzed by western blots in EAC cell lines (FLO1, OE33 and JH-1) under ectopic expression of GLI1. HSP90 protein was used as a loading control (*N* = 3). (**D**) mRNA expression levels of GLI1 were determined in EAC cell lines infected with shRNA against GLI1 and were normalized to the relative expression values of the control (*N* = 3). (**E**) Colony formation assays were performed in EAC cell lines under GLI1 knocking down during two weeks. (**F**) The quantification of the colony number in EAC cell lines infected with shRNA against GLI1 was normalized to the control (*N* = 3). (**G**) Cell viability assays were performed in EAC cell lines after knocking down of GLI1 (*N* = 3). The RLU were normalized to cell number and were compared to the control.

Since GLI1 transcription factor is aberrantly expressed in EAC and behaves as a final effector controlling specific oncogenic target genes of HH signaling, we evaluated whether GLI1 was required for EAC viability. Increased colony formation ability (Figure 3A) as well as high levels of HH target gene expression and proteins were observed in EAC cell lines under ectopic expression of *GLI1* (Figure 3B–3C). In addition, shRNA knockdown of *GLI1* in all tested EAC cell lines reduced tumor cell viability and their ability to form colonies (Figure 3D–3G). Collectively, these results highlight the important role that GLI1 plays in the maintenance of EAC cell lines.

### EAC cell lines are SMO-dependent but insensitive to vismodegib

**Figure 4:**
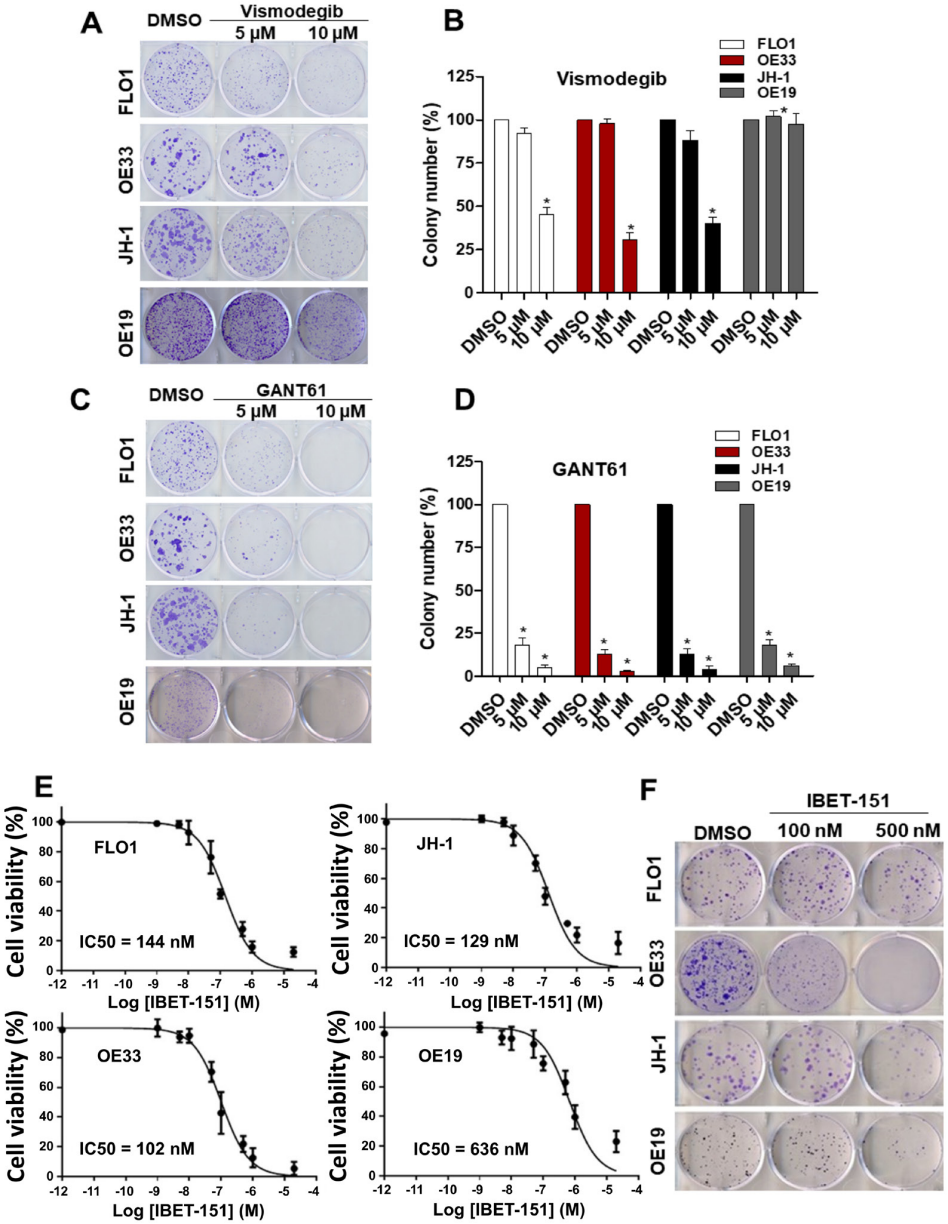
Effect of small molecule inhibitors in EAC. Colony formation assays were performed in EAC cell lines under treatment of two different concentration (5 μM and 10 μM) of SMO (vismodegib) (**A** and **B**) or GLI1 (GANT61) (**C** and **D**) inhibitors (*N* = 3). (**E**) Dose-response curves for cell viability assessed in EAC cell lines exposed to different concentrations of IBET-151 during 48 h (*N* = 3). The RLU were normalized to cell number and were compared to the control (DMSO). (**F**) Colony formation assays were performed in EAC cell lines under treatment of two different concentration (100 nM and 500 nM) of IBET-151, the colony number was quantified and compared to the control (DMSO) (*N* = 3).

Since SMO is a pivotal regulator of GLI signaling, for which a number of small molecule inhibitors are currently approved by the FDA, we evaluated the dependency of EAC cell lines on SMO activity. We infected EAC cell lines with SMO-specific shRNA or control shRNA (Supplementary Figure 2A). Consistent with EAC viability being HH ligand-dependent, *SMO* shRNA reduced tumor cell viability and ability of all four EAC cell lines to grow in a colony formation assay (Supplementary Figure 2B–2D). To take advantage of the dependence of EAC cell lines on SMO and GLI1 activity, we next treated EAC cell lines with small molecule inhibitors of either SMO (vismodegib) or GLI1 (GANT61). While 5 μM GANT61 treatment was able to significantly reduce the growth of EAC cell lines in a colony formation assay, vismodegib had some inhibitory effect on EAC cell lines only at a higher concentration (Figure 4A–4D). Thus, although the expression of *SMO* is required for EAC colony formation, it does not sensitize EAC cells to vismodegib. In contrast, the GLI inhibitor GANT61 was able to attenuate EAC growth, which is consistent with the important role of GLI1 in EAC viability. We also evaluated the effect of vismodegib on the growth of OE33 and OE19 cell line xenografts. There was no significant difference in tumor growth (Supplementary Figure 3A and 3B) and HH target genes expression in both EAC xenografts (Supplementary Figure 3C and 3D) treated with vismodegib as compared to a vehicle group. The reason behind this relative insensitivity to vismodegib is unclear. However, we do note that similarly high IC_50_ values of vismodegib have been reported for a number of other cancer cell lines sensitive to SMO knockdown [22].

### GLI-dependent EAC cell lines are susceptible to inhibition by IBET-151

Although GANT61 is a useful tool compound to demonstrate EAC GLI dependency *in vitro*, it has limited utility *in vivo* and has not been developed for clinical use [23]. Another class of small molecules that has been shown to attenuate GLI activity *in vivo* is BET inhibitors, a number of which are also being evaluated in the clinic. Therefore, we evaluated the effect of IBET-151 treatment on the growth of EAC cell lines using cell viability and colony formation assays. IBET-151 attenuated both cell viability and colony formation of all EAC cell lines tested, with IC_50_ values ranging between 102–636 nM (Figure 4E and 4F). Consistent with our previous work where we had shown IBET-151 acting on GLI1 proteins downstream of SMO [18], IBET-151 also reduced the expression of GLI1 target genes in FLO1 and OE33 EAC cell lines (Supplementary Figure 4A–4C). GLI1 protein levels were also reduced in a dose-dependent manner upon treatment with IBET-151 (Supplementary Figure 4D and 4E), and this observation is consistent with previous studies showing IBET-151 attenuating the activity of a number of genes important to the proliferation and survival of cancer cells, including GLI1 [18]. These results suggest that IBET-151 may affect EAC cell growth via its effects on GLI1. To demonstrate IBET-151 effects on EAC via GLI1, we compared the cell growth and IBET-151 sensitivity of OE33 EAC cell line stably expressing exogenous *GLI1* with those of OE33 expressing a control plasmid. OE33 cells were treated with two different concentrations of IBET-151 (100 nM and 500 nM) for 48 h followed by GLI1 protein and mRNA quantification and a colony formation assay (Supplementary Figure 5A–5C). We observed that OE33 cells expressing exogenous *GLI1* were more resistant to IBET-151 relative to the control cells and had no decrease in GLI1 protein or mRNA levels. These observations are consistent with IBET-151 acting on GLI transcription to attenuate the growth of EAC cells.

### IBET-151 reduces BRD4 occupancy on the GLI1 locus in EAC cells

**Figure 5:**
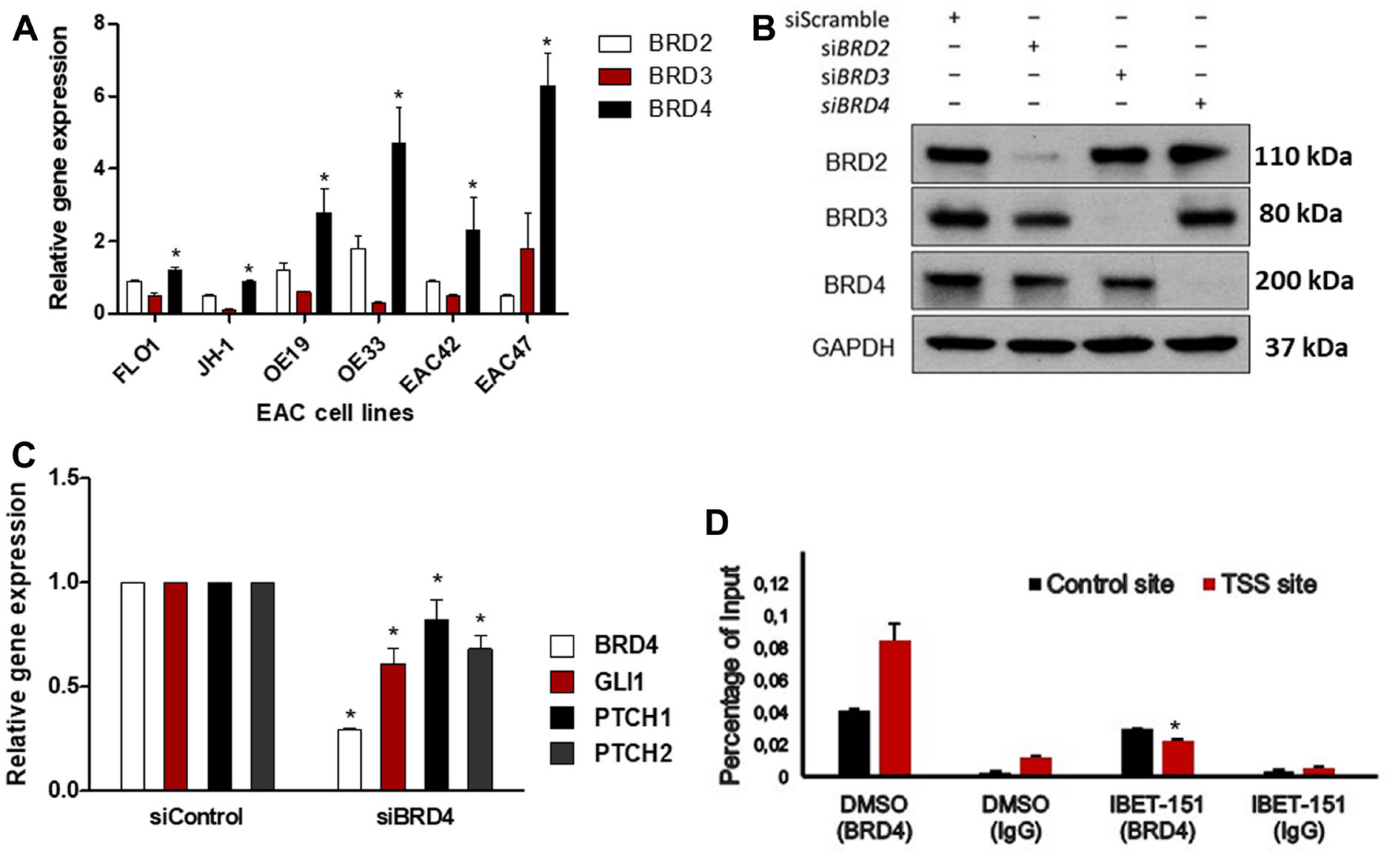
IBET-151 reduces BRD4 occupancy of the GLI1 locus. (**A**) mRNA levels of BRD2, BRD3, or BRD4 genes expression were analyzed and normalized to HPRT (*N* = 3). Statistical significance was indicated by an asterisk to highlight when the BRD4 expression was compared to BRD2 or BRD3 gene expression (**B**) The levels of BRD2, BRD3 or BRD4 protein expression were analyzed by western blots in OE33 cell line under downregulation of *BRD2, BRD3*, or *BRD4* by siRNA (*N* = 3). The level of GAPDH was used as a loading control. (**C**) mRNA levels of HH target genes were evaluated in OE33 cell line under downregulation *BRD4* by siRNA and were normalized to the control (siControl) (*N* = 3). (**D**) BRD4 occupancy was analysed by ChIP-quantitative PCR in OE33 cell line treated with 102 nM of IBET-151 and was normalized to a control ChIP performed using rabbit IgG (*N* = 3). Error bars represent the S. E. of three independent experiments. *p* values ≤ 0.05 are considered statistically significant and indicated by an asterisk (^*^).

**Figure 6:**
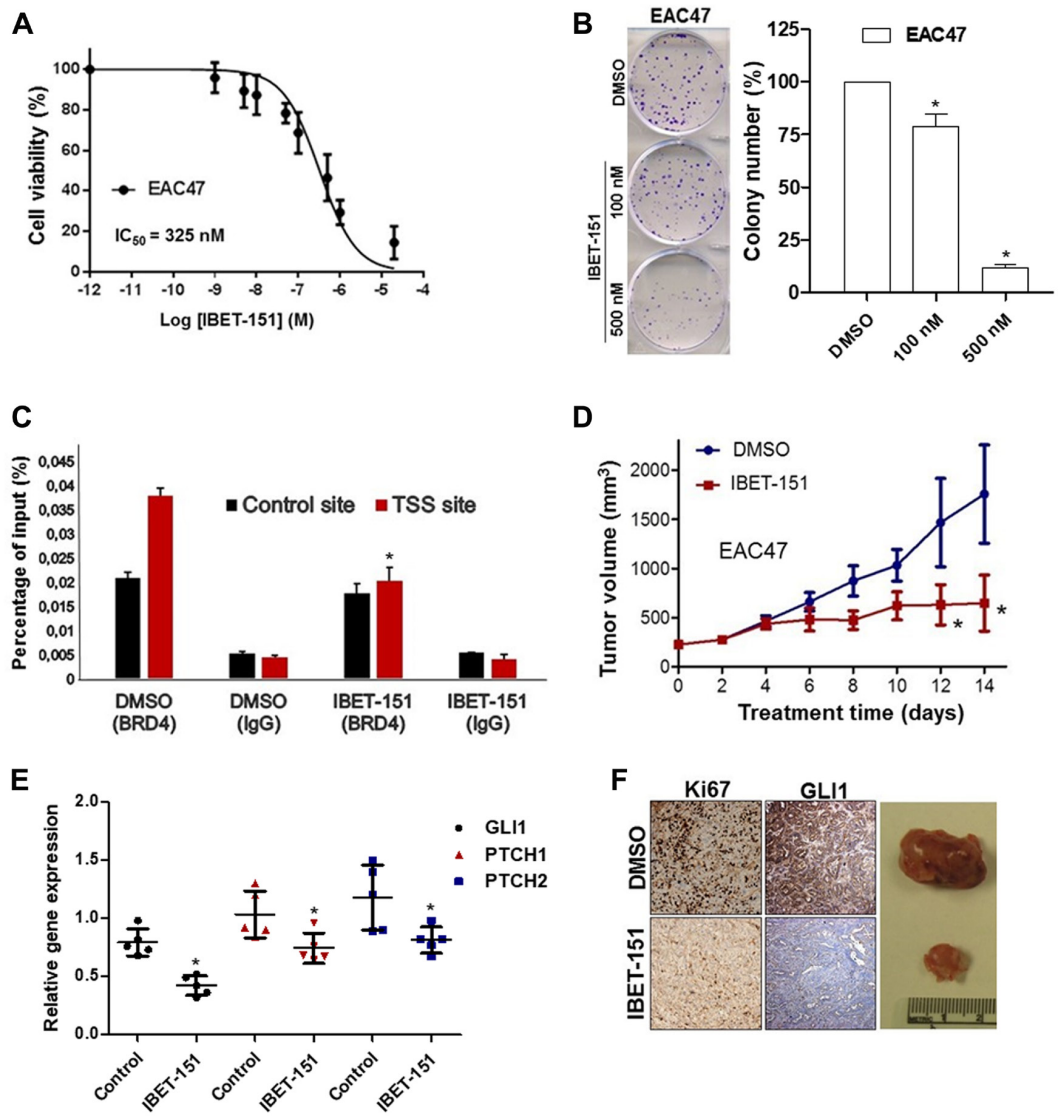
IBET-151 inhibits the growth in EAC PDX models. (**A**) Dose-response curve for cell viability assessed in EAC47 cell line exposed to different concentrations of IBET-151 during 48 h (*N* = 3). (**B**) Colony formation assays were performed in EAC cell lines under treatment of two different concentration (100 nM and 500 nM) of IBET-151, the colony number was quantified and compared to the control (DMSO) (*N* = 3). (**C**) BRD4 occupancy was analysed by ChIP-quantitative PCR in EAC47 cell line treated with 325 nM of IBET-151 and was normalized to a control ChIP performed using rabbit IgG (*N* = 3). (**D**) Significant changes were observed in the volume of EAC47 derived PDX tumors (*N* = 6) under IBET-151 (30 mg/Kg) daily IP treatment. (**E**) mRNA levels of HH target genes (*GLI1*, *PTCH1* and *PTCH2*) were determined by qRT-PCR in PDX EAC47 and were normalized to the expression value of HPRT (*N* = 5). (**F**) Representative images of Ki67 and GLI1 staining in EAC47 derived PDX tumors treated by IBET-151(down) and vehicle (up) (*N* = 5). Error bars represent the S. E. of three independent experiments. *p* values ≤ 0.05 are considered statistically significant and indicated by an asterisk (^*^).

Since IBET-151 acts as an inhibitor of BR2D, BRD3 and BRD4 proteins, we examined the expression of these *BRD* genes in EAC cells (Figure 5A). All three genes were expressed at different levels depending on the EAC cell line used, with relative *BRD4* expression being the highest in OE33 and EAC47. In order to evaluate the dependency of *BRD4* on GLI1 activity, OE33 cell line was transfected with either *BRD4* siRNA or control siRNA (Figure 5B). *BRD4* siRNA reduced the expression of GLI-dependent HH target genes (*GLI1*, *PCTH1* and *PTCH2*) as compared to control siRNA (Figure 5C). Next, we tested the hypothesis that IBET-151 treatment attenuates GLI transcription by reducing BRD4 occupancy on the *GLI1* locus. We used OE33 cell line because it exhibited high BDR4 expression levels. We found that BRD4 associates with the proximal regulatory region of the *GLI1* locus near the transcriptional start site (TSS) (Figure 5D). We also used EAC47 cells derived from a resected patient EAC since these cells exhibited the highest BDR4 expression levels of all the EACs examined. IBET-151 treatment attenuated the cell viability and colony formation ability of EAC47 cell line (Figure 6A and 6B). In addition, IBET-151 decreased the occupancy of BRD4 near the TSS of the *GLI1* locus in EAC47 cells (Figure 6C). These results suggest that IBET-151 abrogates GLI transcription by reducing the association of BRD4 with the proximal regulatory region of the *GLI1* locus in EAC cell lines.

### IBET-151 inhibits EAC growth in patient-derived xenograft models

PDX models retain the complexity and heterogeneity of primary human cancers, and thus are thought to be more clinically relevant when used to evaluate novel small molecules. Based on this rationale, we implanted three different patient-derived EACs (EAC47, EAC36 and EAC42) into the flanks of immunocompromised female mice. Once the tumors reached ~200 mm^3^, the mice were treated with IBET-151 (30 mg/kg) via daily intraperitoneal injections for 14 days. Tumor growth and GLI-dependent HH target genes of all three PDX models were significantly attenuated in the IBET-151 treatment group as compared to the vehicle-treated group (Figure 6D and 6E; Supplementary Figure 6A–6F). In comparison to the control mice, IBET-151 treatment also reduced the levels of a proliferation biomarker (Ki67) and the expression of *GLI1* in EAC47 PDX (Figure 6F). These results show that IBET-151 also significantly abrogated patient-derived EAC growth *in vivo* in part via attenuation of GLI signaling.

## DISCUSSION

Previous studies have reported aberrant activation of HH/GLI signaling in esophageal cancer, which can be caused by a number of factors [24–26] including genetic alterations of individual pathway components, such as *PTCH* or *SMO* mutations and *GLI1/2* amplification [27]. The over-expression of GLI1 is a credible indicator of poorer prognosis for a variety of cancers including gastric cancer and esophageal squamous cell carcinoma (ESCC) [28, 29]. To date, the correlation between the expression of GLI1 and its clinical significance in EAC has not been reported. In the present study, we demonstrate that GLI1 is over-expressed in the primary EAC tumors derived from a group of patients as well as in EAC cell lines. This aberrant expression of GLI1 was associated with high expression levels of other HH target genes, such as *PTCH1, HHIP*, and *MYCN.* We also observed that GLI1 played a role in EAC response to NAC, which suggests that GLI1 expression in tumor may predict patients’ response to chemotherapy. Since EAC cell lines with ectopic expression of GLI1 had increased expression levels of HH target genes and GLI1 depletion decreased the tumor ability to form colonies, we conclude that GLI1 is a critical regulator of EAC maintenance. The upregulation of downstream effectors in the HH signaling cascade such as GLI1 has been suggested as a possible mechanism to explain tumor resistance to the SMO inhibitors [14, 30], as is the case with vismodegib. Although the high levels of GLI1 in EAC are consistent with HH-driven tumors reports, our data did not allow determining whether EAC cells are inherently resistant to vismodegib as a consequence of aberrant activation of GLI1. Another possible contributor to such a resistance is Notch signaling. Previously, we reported the same EAC tumors and cell lines evaluated in this study having elevated Notch activity, which is associated with the differentiation state and clinical stage of EAC, drives resistance to chemotherapy, and results in poor prognosis [32]. Furthermore, elevated Notch activity has been related with resistance in HH-driven BCC [31]. Together, these results suggest that aberrant activity of Notch signaling may contribute to the resistance to vismodegib in HH-driven EAC.

Two SMO inhibitors have been approved for treating advanced BCC, vismodegib [11] and sonidegib [12]. However, the evidence of inherent and acquired resistance to SMO inhibition has been reported in the clinic [33, 34]. Currently, there is no FDA-approved HH inhibitor for EAC patients. However, there is an ongoing clinical trial with Itraconazole, a repurposed anti-fungal agent that acts as a SMO inhibitor [35]. The purpose of this study is to demonstrate that orally administered Itraconazole can inhibit HH signaling in patients with esophageal cancer, EAC and ESCC [35]. Pathway-dependent genetic alterations discovered in resistant tumors from patients and animal models directly affect HH pathway members [36]. Initial studies in medulloblastoma and BCC suggest that vismodegib resistance stems from genetic alterations at the level of or downstream from SMO [37–39]. Resistance can originate from SMO point mutations that ablate SMO–drug interaction while maintaining HH pathway activation [40]. Similarly, in esophageal cancer resistant to SMO inhibitors, the activity of PI3K/Akt/mTOR pathway component S6 kinase 1 (S6K1) was found to be elevated [15]. S6K1 phosphorylates GLI1 releasing it from Sufu inhibition to activate GLI-dependent transcription. S6K1 renders GLI1-expressing tumors partially SMO-independent as inhibition from Sufu is derepressed. Interestingly, S6K1 is inappropriately activated in EAC and some medulloblastomas [15, 41], providing a partial explanation for drug resistance [42]. Developing effective targeted therapies to dispatch tumors before they evolve resistance requires knowledge of available escape pathways. This is especially critical given that resistant clones are likely present in small numbers at the time of treatment initiation. GLI transcription factors ultimately transduce the signal from the HH ligand. Moreover, escape pathways that bypass SMO still activate GLI1. Targeting GLI1 directly or the signaling components that activate GLI1 may prove quite successful as the next level of therapy.

Since the resistance to SMO inhibitors, such as vismodegib, occurs via genetic changes of SMO or other downstream HH components, we propose to overcome these resistance mechanisms by modulating GLI transcription via BET bromodomain proteins inhibition. Specifically, IBET-151 attenuates HH signaling in cells with constitutive GLI1 activity in a SMO-independent fashion in BCC, medulloblastoma, and atypical teratoid/rhabdoid tumor [15, 17, 18]. Given that vismodegib-resistant EAC cells show aberrant activation of GLI1, we hypothesized that IBET-151 treatment may bypass this vismodegib resistance. Our results show that IBET-151 has a potential effect in EAC cell lines resistant to vismodegib inhibiting cell viability, ability to form colonies, and GLI1 target gene expression. Additionally, it has been reported that BRD4 regulates *GLI* transcription downstream of SMO and SUFU, and ChIP studies reveal that BRD4 directly occupies *GLI1* and *GLI2* promoters [15, 17, 18]. Our results are consistent with these data, as we show that BRD4 associates with the proximal regulatory region of the GLI1 locus near its transcriptional start site. Simultaneously, we demonstrated that IBET-151 treatment attenuates GLI1 transcription by reducing BRD4 occupancy on the *GLI1* locus in both EAC47 cells derived from a patient tumor and OE33 cell line. In order to extend our findings, we evaluated the effect of IBET-151 in EAC-PDX samples. We demonstrated that IBET-151 attenuated the tumor growth from three different EAC-PDX models when compared to the vehicle group. Moreover, IBET-151 reduced both the expression of *GLI1* and proliferation of EAC47 PDX. Although the majority of HH inhibitors described to date bind to and attenuate the activity of SMO, downstream mutations from SMO or other signaling pathways activate GLI proteins in a non-canonical manner contributing to the resistance to SMO inhibitors. We provide a viable mechanism where IBET-151 treatment abrogates the growth of vismodegib-resistant and GLI1-dependent EAC cells, downregulating the levels and activity of the GLI1 transcription factor by reducing BRD4 occupancy on the *GLI1* locus. Currently, several BET inhibitors are in different stages of clinical evaluation and will provide the first starting point for future therapies targeting GLI-dependent EAC [43, 44].

## MATERIALS AND METHODS

### Human esophageal adenocarcinoma and normal esophageal mucosa samples

Human EAC tumors, matched adjacent non-tumor tissues, and normal esophageal mucosa were obtained from tissue microarray (Biomax. US, ES8011) and the patients undergoing surgery (ultrasound-assisted endoscopic [EUS] biopsies) at Miller School of Medicine, University of Miami. We obtained consent from all patients and approval from the Institutional Research Ethics Committee. We analyzed the expression of GLI1 in 22 chemo-naive EAC samples derived from ultrasound-assisted endoscopic biopsies and compared to the response of NAC therapy (FOLFOX).

### Cell culture

Human EAC cell lines OE33 and OE19 were obtained from the European Collection of Cell Culture. FLO1 and JH-EsoAd1 cells were a generous gift from other labs [32]. Human EAC36, EAC42, and EAC47 cell lines were isolated from human esophageal mucosa obtained from tumor adjacent tissue. Normal human primary esophageal epithelial cells EAC36N, EAC42N, and EAC47N were isolated from human esophageal mucosa obtained from normal adjacent tissue. All cell lines were characterized by short tandem repeat analyses (STR) profiling (LGS Standards SLU) within 6 months after receipt.

### Compounds

Commercially available compounds were purchased from either Selleckchem or R & D system as individual compounds. GDC-0449 (S1082; Selleckchem), GANT61 (S8075; Selleckchem) and IBET-151 (4650; R & D System).

### Colony formation and cell viability assays

Cells were cultured at low density under treatment, and then colonies were stained with 0.01% crystal violet and counted. The cells were measured using the Cell Titer-Glo assay (G7572; Promega) for Cell Viability Assays.

### qRT-PCR analysis

Total mRNA was isolated and cDNA was synthesized according to the manufacturer’s protocol (4368814; Life Technologies). qRT-PCR analysis was performed using TaqMan probes according to manufacturer’s instructions (4324018; Applied Biosystems). Gene expression was normalized to HPRT or GAPDH gene. TaqMan Primer sequences are available upon request.

### Transfection of adherent cells

The specific siRNA duplexes targeted against human BRD2, BRD3, or BRD4, siRNA transfection reagent, and reduced-serum transfection medium were purchased from Santa Cruz Biotechnology (Santa Cruz, CA, USA). The day before transfection, 4 × 10^4^ cells were seeded in each well of 12-well cell culture plates in RPMI 1640 medium containing 10% FCS without antibiotics and incubated for 24 h. The next day, transfection complexes were prepared using BRD siRNA, siRNA transfection reagent, and transfection medium according to the manufacturer’s instructions and were delivered to cell monolayers in 600 μl fresh media with 50 or 100 nM final concentration of siRNA duplexes. A scrambled siRNA (Santa Cruz Biotechnology) was used as negative control.

### Lentiviruses and infection

Lentiviruses expressing various shRNAs and over-expression plasmids were produced as described previously [18]. For viral infection, sub-confluent cells were overlaid with the virus-containing medium and fresh growth medium in the presence of polybrene (H9268; Sigma).

### Western blotting

Cells lysates were resolved by SDS-PAGE and transferred onto Immobilon-P membranes (IPVH00010; Millipore). Membranes were blocked in milk and incubated with primary antibodies (BRD2 mAb #5848; BRD3 SC-515666; BRD4 mAb #13440; GLI1 mAb #3538; HSP90 mAb #4877; GAPDH mAb #5174) followed by incubation with the anti-mouse or anti-rabbit secondary antibody conjugated with horseradish peroxidase. For detection, enhanced chemiluminescence reaction (RPN2232; Amersham Biosciences) was performed according to the manufacturer’s specifications.

### Immunohistochemistry

Immunohistochemical staining of GLI1 (1:200; ab-151796) and Ki67 (1:100; ab-15580) was carried out using a DAKO autostainer. Results were scored using Semiquantitative Scoring (IRS) by multiplying the percentage of positive cells (P) by the intensity (I). Formula: Q = P × I.

### Chromatin immunoprecipitation (ChIP) assay

ChiP experiments were performed as described previously [18].

### Animal experiments

Since the therapeutic efficacy in PDX models is a better predictor of the clinical response from patient tumors, we used three EAC PDX (EAC36, EAC42 and EAC74) from our library of surgically resected patient tumors in order to evaluate the effect of IBET-151 on tumor growth. Six-week-old NOD-SCID gamma (NSG) female mice were purchased from Jackson Laboratories. PDX cancer models and xenografts were established as described previously in NSG mice [45]. When the tumor size reached 200 mm^3^, the mice were treated with daily dose of 30 mg/kg by intraperitoneal (IP) injection (5 mice per treatment/5 mice per negative control) during 14 days of treatment. The stop/end point criteria are based on the days it takes for tumors to reach 2000 mm^3^ according to what is approved in our animal protocol. Tumor volume was measured by the formula: volume = (*S* × *S* × *L*)/2 [43]. The tumor samples were subjected to histologic examination and qRT-PCR analysis. Animal experiments were reviewed and approved by the University of Miami Institutional Animal Care and Use Committee (Miami, FL, USA).

### Statistic

*P* value was calculated using chi-square in the contingency table. Data are presented as mean ± SD and were analyzed by 2-tailed Student’s *t* test. A *P* value of less than 0.05 was considered significant. Enhanced expression of GLI1 in EAC tumors versus normal mucosa was determined by the Mann–Whitney *U* Test. In all other cases, statistical significance was determined by Student’s *T* test. *P* value < 0.05 was considered statistically significant.

## SUPPLEMENTARY MATERIALS



## Abbreviations

BCC: basal cell carcinoma
*BCL2*: B-cell lymphoma 2
BET: bromodomain and extra-terminal motif
BRD: bromodomain
ChIP: Chromatin Immunoprecipitation
EAC: Esophageal adenocarcinoma
EMT: epithelial–mesenchymal transition
ESCC: esophageal squamous cell carcinoma
EUS: ultrasound-assisted endoscopic
FDA: food and drug administration
GAPDH: glyceraldehyde-3-phosphate dehydrogenase
GLI: Glioma-associated oncogene homolog
HH: Hedgehog
*HHIP*: hedgehog-interacting protein
HPRT: hypoxanthine guanine phosphoribosyl transferase
IHC: immunohistochemistry
IRS: the immunoreactive score
*MMP9*: Matrix metallopeptidase 9
MTOR: *mammalian target of rapamycin*
NAC: Neoadjuvant chemotherapy
NSG: NOD-SCID gamma
*PDGFR*: *Platelet-derived growth factor receptors*
PDX: patient-derived xenograft
PI3K: Phosphoinositide 3-kinases
PTCH: Patched
qRT-PCR: Quantitative reverse transcription polymerase chain reaction
S6K1: Ribosomal protein S6 kinase beta-1
SDS-PAGE: sodium dodecyl sulphate-polyacrylamide gel electrophoresis
SMO: Smoothened
STR: short tandem repeat
TCGA: the cancer genome atlas
TSS: transcription start site(s)
*VEGF*: *Vascular endothelial growth factor*
