# Supplementary material for: The bromodomain inhibitor IBET-151 attenuates vismodegib-resistant esophageal adenocarcinoma growth through reduction of GLI signaling
Source: Oncotarget. 2020 Aug 18;11(33):3174–87. doi: 10.18632/oncotarget.27699 (PMC7443367; doi:10.18632/oncotarget.27699)
Supplement: Supplementary file 1 [file oncotarget-11-3174-s001.pdf]

The bromodomain inhibitor IBET-151 attenuates vismodegib-resistant esophageal adenocarcinoma growth through reduction of GLI signaling

SUPPLEMENTARY MATERIALS

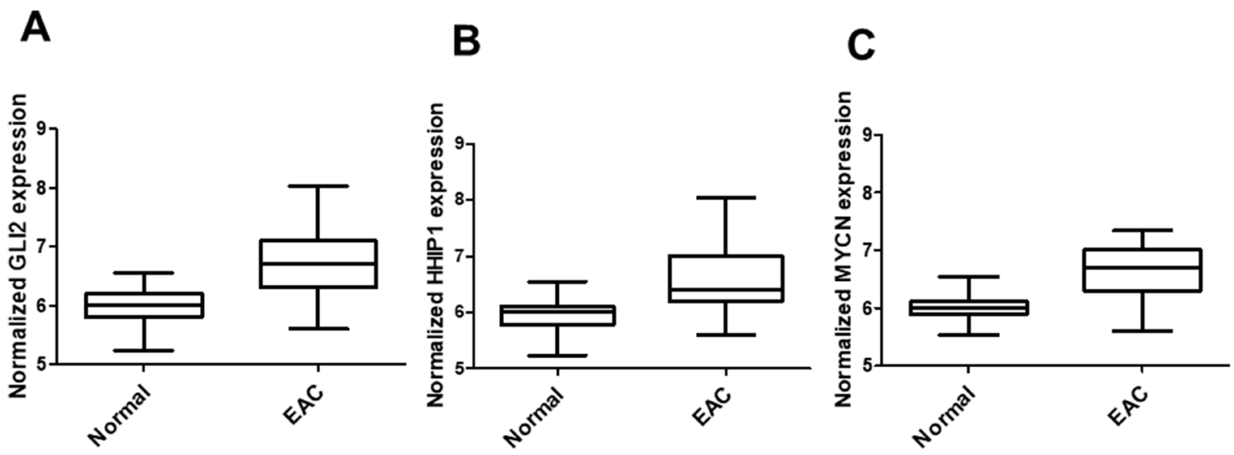

Supplementary Figure 1: GLI signaling biomarkers from TCGA RNAseq are elevated in EAC. Boxplots depicting RNA levels for *GLI2* (**A**), *HHIP1* (**B**) and *MYCN* (**C**) from 103 patients' samples (EAC tumors (n = 75), adjacent normal mucosa (n = 28). p values $\leq$ 0.05 are considered statistically significant and indicated by an *asterisk*.

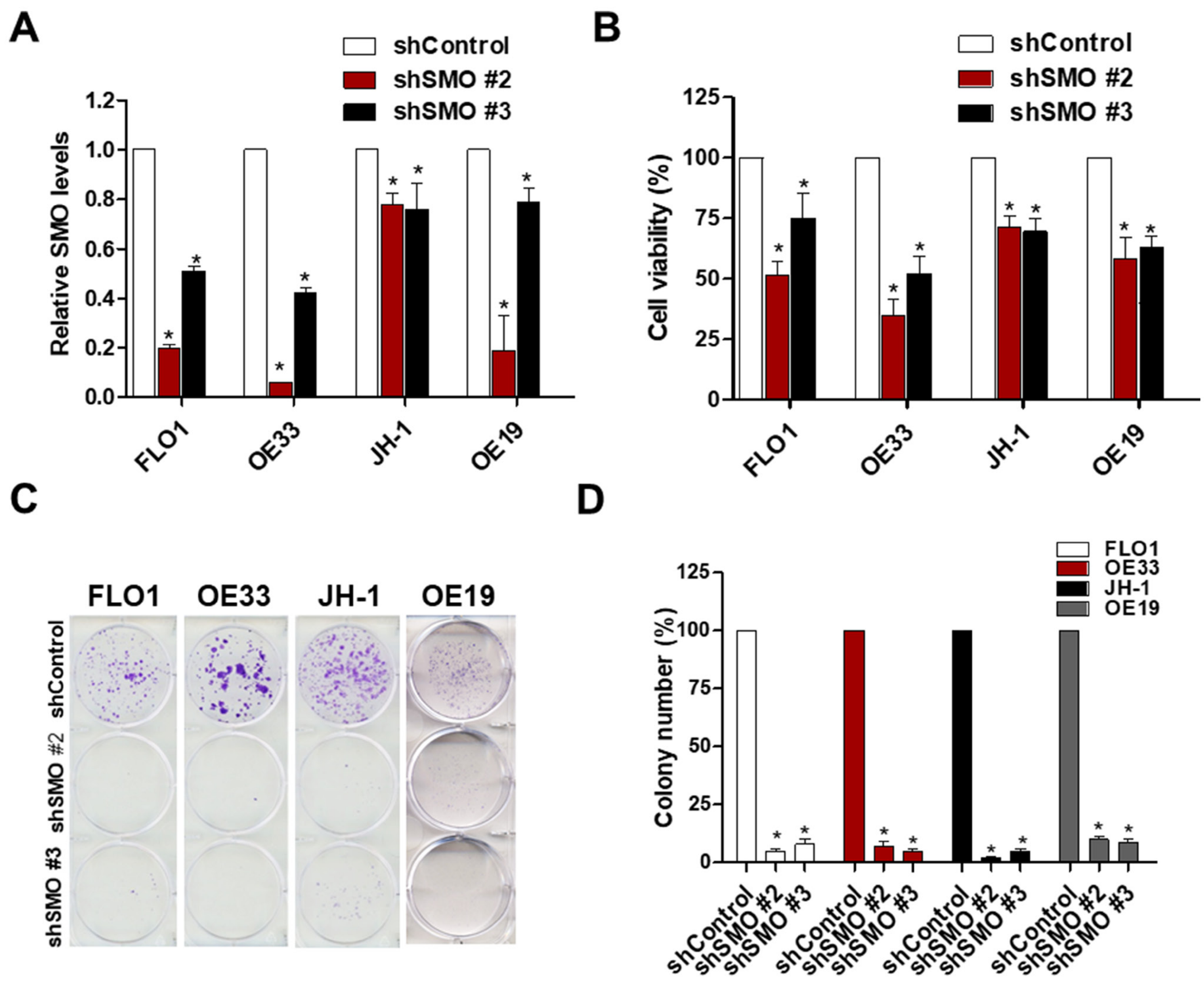

Supplementary Figure 2: Dependency of EAC cell lines on SMO activity. (**A**) mRNA expression levels of SMO were determined in EAC cell lines infected with shRNA against SMO ($N = 3$). (**B**) Cell viability assays were performed in EAC cell lines after knocking down of SMO. The RLU were normalized to cell number ($N = 3$). (**C–D**) Colony formation assays were performed in EAC cell lines under SMO knocking down during two weeks ($N = 3$).

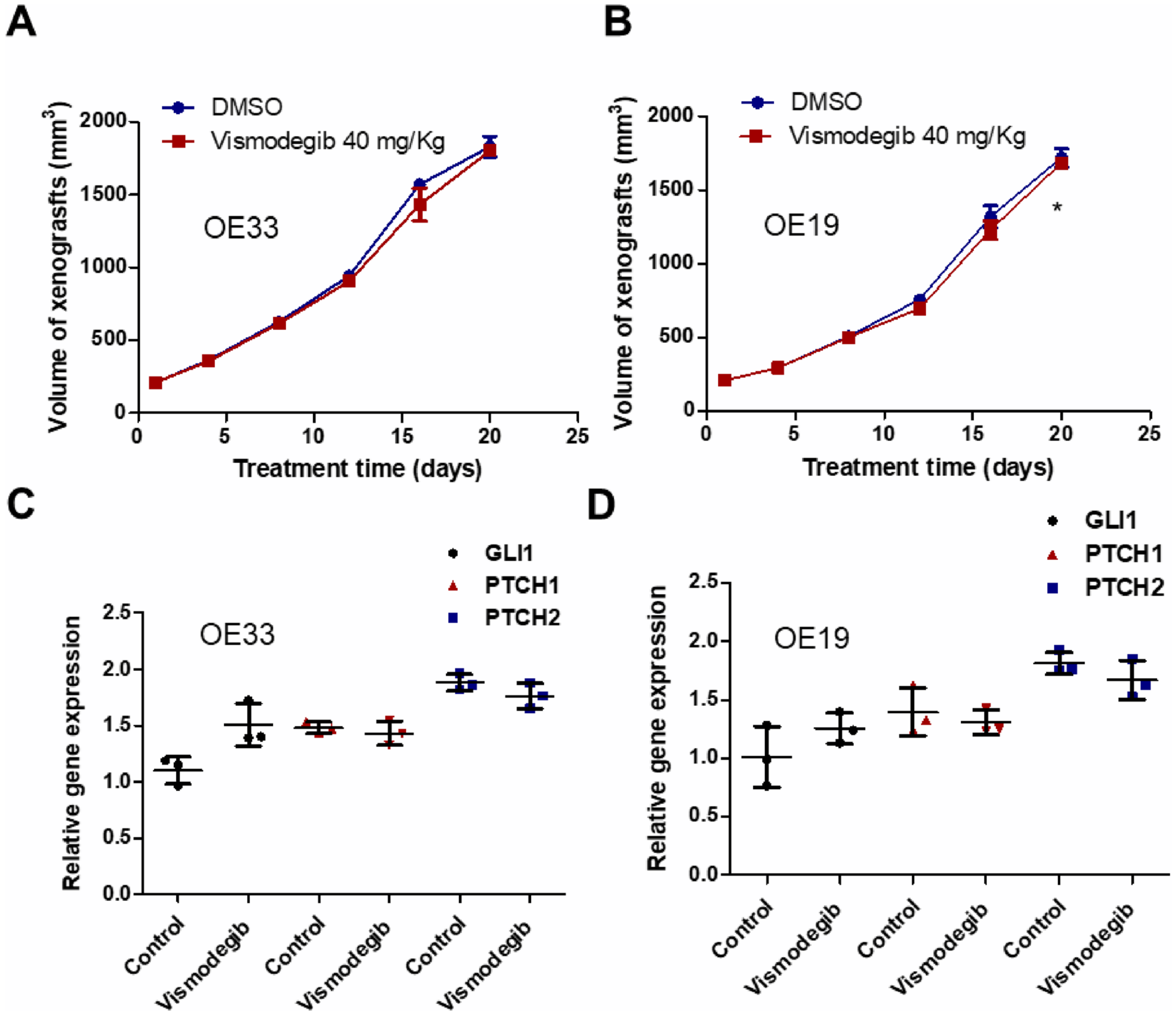

Supplementary Figure 3: EAC cell lines are insensitive to Vismodegib. No significant changes were observed in the volume of xenografts derived from OE33 (**A**) and OE19 (**B**) cell lines under vismodegib (40 mg/Kg) daily IP treatment (*N* = 3). mRNA levels of HH target genes (*GLI1*, *PTCH1* and *PTCH2*) were determined by qRT-PCR in OE33 (**C**) and OE19 (**D**) derived from xenografts under vismodegib (40 mg/Kg), and were normalized to the expression value of HPRT (*N* = 3). Error bars represent the S.E. of three independent experiments. *p* values ≤ 0.05 are considered statistically significant and indicated by an *asterisk*.

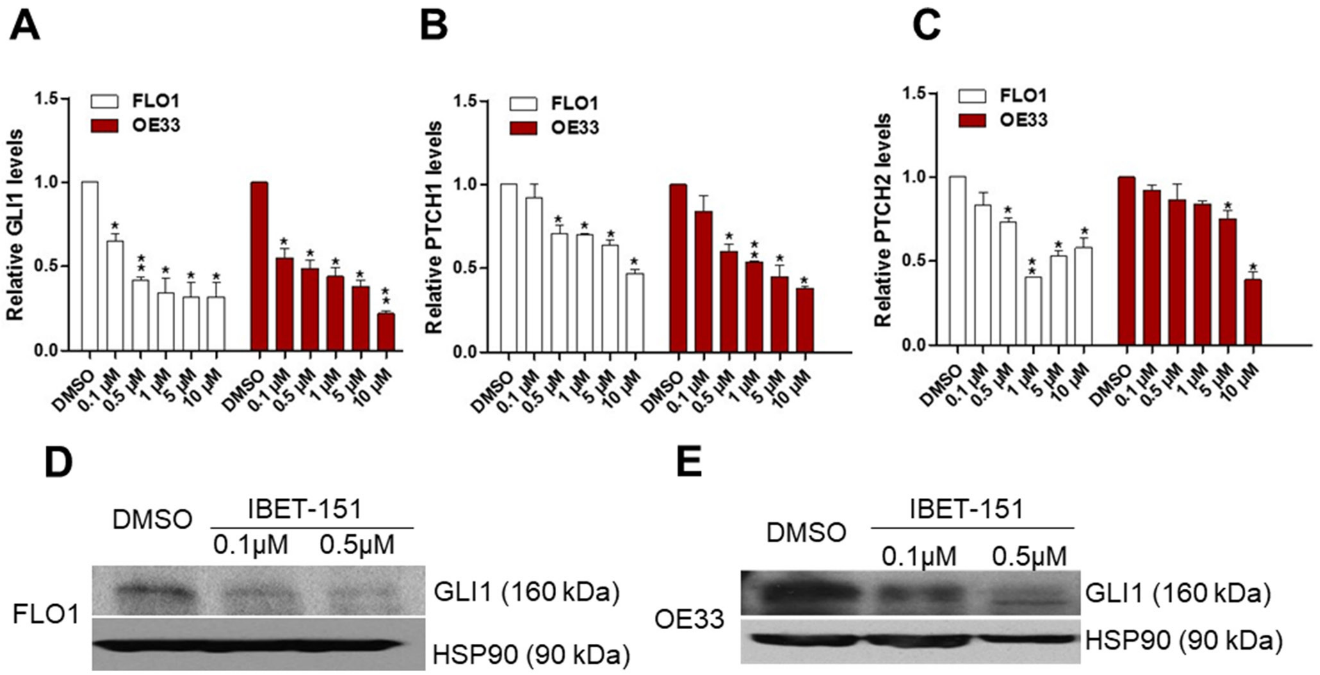

Supplementary Figure 4: Effect of IBET-151 in EAC cell lines. (**A–C**) mRNA expression levels of HH target genes (*GLI1*, *PTCH1* and *PTCH2*) were determined in FLO1 or OE33 EAC cell lines and were normalized to HPRT (*N* = 3). The effect of IBET-151 on GLI1 gene products in FLO1 (**D**) or OE33 (**E**) cell lines was examined through western blot analyses (*N* = 3). The level of HSP90 was used as a loading control.

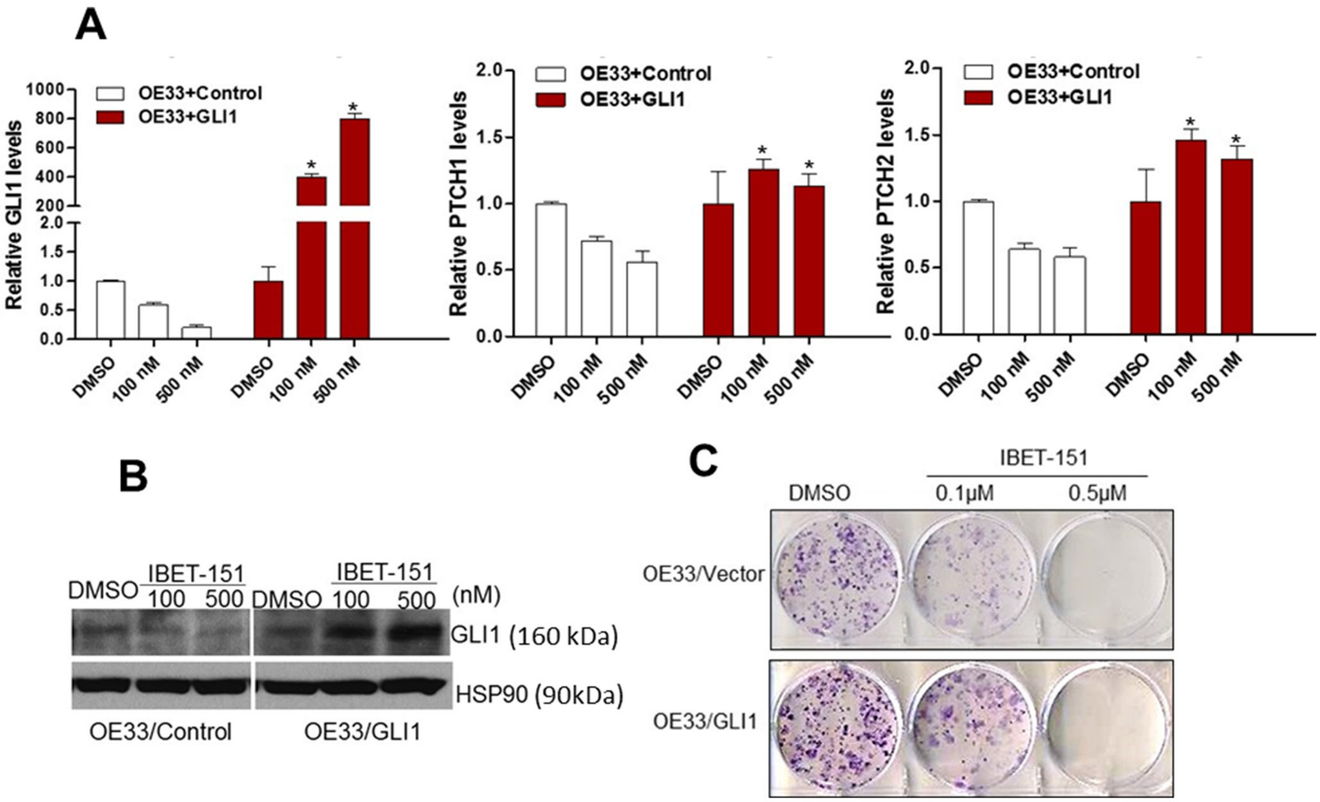

Supplementary Figure 5: Effect of IBET-151 in OE33 cell line expressing exogenous GLI1. mRNA expression levels of HH target genes (*GLI1*, *PTCH1* and *PTCH2*) were determined in OE33 (**A**) EAC cell line under ectopic expression of GLI1 and were normalized to HPRT ($N = 3$). (**B**) The effect of IBET-151 on GLI1 gene products in OE33 cell line was examined through western blot analyses ($N = 3$). The level of HSP90 was used as a loading control. (**C**) Colony formation assays were performed in OE33 cell line under ectopic expression of GLI1 treatment of two different concentration (100 nM and 500 nM) of IBET-151 ($N = 3$). Error bars represent the S.E. of three independent experiments. p values ≤ 0.05 are considered statistically significant and indicated by an *asterisk*.

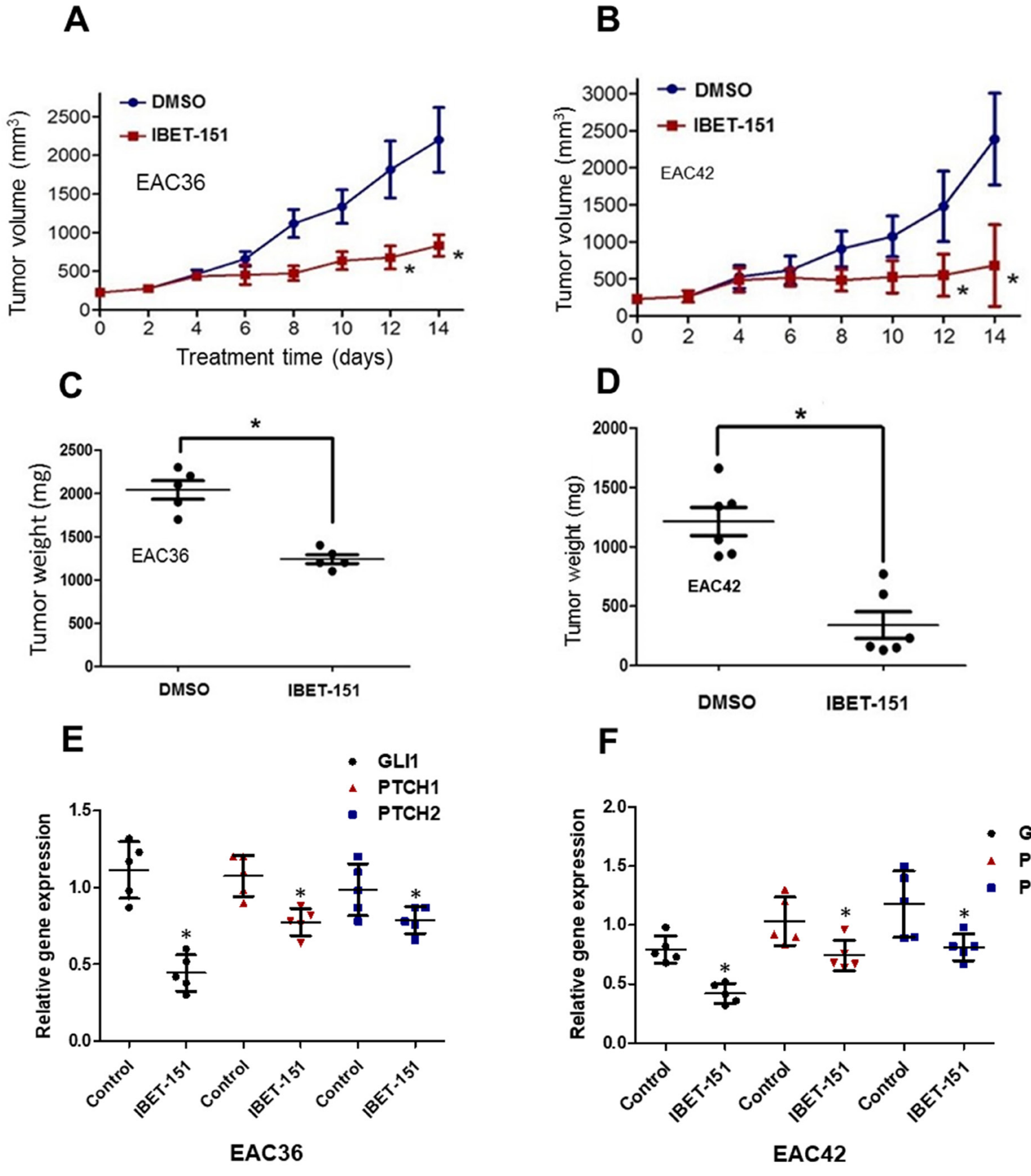

Supplementary Figure 6: IBET-151 inhibits tumor growth in EAC PDX models. (**A–B**) Significant changes were observed in the volume of EAC derived PDX tumors ($n = 6$) under IBET-151 (30 mg/Kg) daily IP treatment. (**C–D**) Tumor weight was evaluated at the end of the experiment ($N = 5$). (**E–F**) mRNA levels of HH target genes (*GLI1*, *PTCH1* and *PTCH2*) were determined by qRT-PCR in EAC PDXs and were normalized to the expression value of HPRT ($N = 5$). Error bars represent the S.E. of three independent experiments. p values ≤ 0.05 are considered statistically significant and indicated by an *asterisk*.
